# In Vivo Biocompatible Self-Assembled Nanogel Based on Hyaluronic Acid for Aqueous Solubility and Stability Enhancement of Asiatic Acid

**DOI:** 10.3390/polym13234071

**Published:** 2021-11-23

**Authors:** Yu Yu Win, Penpimon Charoenkanburkang, Vudhiporn Limprasutr, Ratchanee Rodsiri, Yue Pan, Visarut Buranasudja, Jittima Amie Luckanagul

**Affiliations:** 1 Department of Pharmaceutics and Industrial Pharmacy, Faculty of Pharmaceutical Sciences, Chulalongkorn University, Bangkok 10330, Thailand; yuyuwin957@gmail.com; 2 Nabsolute Co., Ltd., Bangkok 10700, Thailand; c.penpimon@gmail.com; 3 Department of Pharmacology and Physiology, Faculty of Pharmaceutical Sciences, Chulalongkorn University, Bangkok 10330, Thailand; vudhiporn.l@pharm.chula.ac.th (V.L.); ratchanee.r@pharm.chula.ac.th (R.R.); visarut.b@pharm.chula.ac.th (V.B.); 4 Preclinical Toxicity and Efficacy Assessment of Medicines and Chemicals Research Cluster, Chulalongkorn University, Bangkok 10330, Thailand; 5 Guangdong Provincial Key Laboratory of Malignant Tumor Epigenetics and Gene Regulation, Guangdong-Hong Kong Joint Laboratory for RNA Medicine, Medical Research Center, Sun Yat-sen Memorial Hospital, Sun Yat-sen University, Guangzhou 510120, China; panyue@mail.sysu.edu.cn; 6 Natural Products for Ageing and Chronic Diseases Research Unit, Chulalongkorn University, Bangkok 10330, Thailand

**Keywords:** asiatic acid, hyaluronic acid, poly(N-isopropylacrylamide), thermoresponsive nanogel, stability study, cytotoxicity study, in vivo animal study

## Abstract

Asiatic acid (AA), a natural triterpene found in *Centalla asiatica*, possesses polypharmacological properties that can contribute to the treatment and prophylaxis of various diseases. However, its hydrophobic nature and rapid metabolic rate lead to poor bioavailability. The aim of this research was to develop a thermoresponsive nanogel from hyaluronic acid (HA) for solubility and stability enhancement of AA. Poly(N-isopropylacrylamide) (pNIPAM) was conjugated onto HA using a carbodiimide reaction followed by ^1^H NMR characterization. pNIPAM-grafted HA (HA-pNIPAM) nanogels were prepared with three concentrations of polymer, 0.1, 0.15 and 0.25% *w*/*v*, in water by the sonication method. AA was loaded into the nanogel by the incubation method. Size, morphology, AA loading capacity and encapsulation efficiency (EE) were analyzed. In vitro cytocompatibility was evaluated in fibroblast L-929 cells using the PrestoBlue assay. Single-dose toxicity was studied using rats. HA-pNIPAM nanogels at a 4.88% grafting degree showed reversible thermo-responsive behavior. All nanogel formulations could significantly increase AA water solubility and the stability was higher in nanogels prepared with high polymer concentrations over 180 days. The cell culture study showed that 12.5 µM AA in nanogel formulations was considered non-toxic to the L-929 cells; however, a dose-dependent cytotoxic effect was observed at higher AA-loaded concentrations. In vivo study proved the non-toxic effect of AA loaded in HA-pNIPAM nanogels compared with the control. Taken together, HA-pNIPAM nanogel is a promising biocompatible delivery system both in vitro and in vivo for hydrophobic AA molecules.

## 1. Introduction

Asiatic acid (AA) is one of the major bioactive triterpenes present in *Centella asiatica*, whose common names are pennywort and gotu koka [1]. AA possesses anti-oxidative, anti-inflammatory [2,3,4,5], anticancer [6,7,8], antifungal [9], antimicrobial [10,11], antidiabetic and antihyperlipidemic activities [12]; and cardiac, renal, hepatic and neuroprotective effects [13,14,15,16,17]. It also has the potential to inhibit osteoporosis [18,19] and fibrotic diseases [13,20,21], and to induce collagen synthesis and wound healing [22]. However, AA is highly lipophilic (log *p* value, 5.7), poorly soluble in water (0.0598 mg/L at 25 °C) and undergoes rapid metabolism by the liver. Although studies have demonstrated the therapeutic activities of AA against many diseases, the limitations of poor bioavailability and rapid metabolism hinder this compound from developing into therapeutic applications [23].

There are several approaches for delivery of insoluble drugs, such as the prodrug strategy [24], pH modification using a pH modifier and salt form, co-solvency, surfactant solubilization, amorphous formation, solid dispersions, cocrystals, nanoparticle delivery systems consisting of polymeric micelles, nanocrystals, nanosuspensions, solid lipid nanoparticles, liposomes, microemulsions and self-emulsifying drug delivery systems [25] and nanogels [26]. There are also several formulations intended to enhance water solubility of AA [27,28,29,30]. Among the nanocarriers, nanogels are one of the promising drug delivery systems.

Nanogels are physically or chemically crosslinked hydrogels in the form of nanoparticles. Nanogels possess a large surface area and high water content. They can deliver both hydrophilic and hydrophobic drugs [31]. Nanogels have the advantages of easy drug loading, high loading capacity, physical stability and a stimuli-responsive nature. Drugs can be loaded into the polymer matrix through electrostatic, van der Waals or hydrophobic interactions resulting in the formation of stable nanoparticles [26,32]. The hydrophilic polymer can be modified with hydrophobic groups to form an amphiphilic polymer, which can self-assemble into nanogel in aqueous condition. Subsequently, the hydrophobic part of the nanogel can encapsulate the hydrophobic drugs by hydrophobic interaction [33].

Hyaluronic acid (HA) is a natural linear polysaccharide derived from the β (1,4) and β (1,3) glycosidic bonding of the two repeated disaccharide units D-glucoronic acid and N-acetyl-D-glucosamine [34]. It is highly hydrophilic, biocompatible and biodegradable and has three functional groups (hydroxyl-, carboxyl- and N-acetyl) available for chemical modifications [35]. Thermoresponsive polymers such as poly(N-isopropylacrylamide) (pNIPAM) and pluronic acid could interact with HA through chemical reaction and the resulting modified HA could be physically crosslinked into hydrogel through hydrophobic interaction [36,37].

pNIPAM is the most widely used thermoresponsive water-soluble polymer and has various applications in the fields of drug delivery systems [36], scaffold in tissue engineering [38] and biosensors [39]. It has both hydrophilic amide (-CONH-) and hydrophobic isoprophyl (-CH(CH_3_)_2_) functional groups in its structure and possesses a lower critical solution temperature (LCST) of 32 °C, above which pNIPAM undergoes coil-to-globule transition due to the dehydration of the polymer chain [40]. As the LCST is close to the body temperature, it transforms into a gel state in the body. Modification of pNIPAM with hydrophilic polymers such as chitosan and hyaluronic acid, or collagen and gelatin, could alter LCST, enhance mechanical strength and improve biocompatibility [41].

pNIPAM has been conjugated to another polymer through amide bond linkages through carbodiimide coupling interactions using 1-(3-dimethylaminopropyl)-3-ethylcarbodiimide hydrochloride (EDC) and N-hydroxysuccinimide (NHS) as crosslinkers. The conjugated copolymers were used to prepare a hydrogel as a delivery system for controlled release, improving solubility and as a cell carrier [36,41,42,43]. pNIPAM-grafted HA (HA-pNIPAM) hydrogel and nanogels have been used in pharmaceutical applications such as tissue adhesion prevention [44] and solubility enhancement of hydrophobic cyclosporin [45] and curcumin [46], respectively.

In this study, an HA-pNIPAM nanogel delivery system was prepared with 0.1, 0.15 and 0.25% *w*/*v* polymer concentrations attempted for enhancing the solubility and stability of AA and for evaluating the physicochemical properties of the three formulations. Furthermore, a cytotoxicity study using in vitro fibroblast L-929 cells and in vivo study using a rat strain, BrlHan:WIST@Jcl (GALAS), were carried out to analyze the biocompatibility and toxicity of the nanogels.

## 2. Materials and Methods

### 2.1. Materials

Asiatic acid (AA) (purity 95%) was obtained from SEPPIC, Normandie, France. Sodium hyaluronate (HA) (MW = 47 kDa) was purchased from Dali company (Wuhan, Hubei, China), amine-terminated poly(N-isopropylacrylamide) (pNIPAM) (Mn = 5.5 kDa) from Sigma-Aldrich, St. Louis, MO, USA, 1-(3-dimethylaminopropyl)-3-ethylcarbodiimide hydrochloride (EDC) from US Biological Life Sciences and N-hydroxysuccinimide (NHS) from Acros Organics, Morris Plains, NJ, USA. Reagents required to prepare the mobile phase such as acetonitrile and methanol were purchased from RCI Labscan, Bangkok, Thailand, and 85% orthophosphoric acid (H_3_PO_4_) from Sigma-Aldrich, St. Louis, MO, USA. All chemical reagents were of analytical grade and all solvents were of high-performance liquid chromatography (HPLC) grade. For the cell culture experiment, mouse L-929 fibroblasts were obtained from the Faculty of Engineering, Chulalongkorn University, Bangkok, Thailand. Dulbecco’s modified Eagle’s medium (DMEM), fetal bovine serum (FBS) and penicillin-streptomycin (10,000 U/mL) were purchased from Gibco, Waltham, MA, USA. PrestoBlue^TM^ cell viability reagent was purchased from Invitrogen Corporation, San Diego, CA, USA. For the animal study, rats, strain BrlHan:WIST@Jcl (GALAS), were bought from Nomura Siam International, Bangkok, Thailand. 

### 2.2. Synthesis of HA-pNIPAM Polymer

HA-pNIPAM polymer was prepared via EDC/NHS reaction following the method from the previous study [42]. Amounts of 0.5 g of HA and 0.345 g of pNIPAM (1:0.05 HA:pNIPAM molar ratio) were dissolved separately in 25 mL of water each and the two solutions were mixed together. Subsequently, NHS and EDC were added into the mixture as catalysts in a 4:1 excess molar ratio to the carboxyl group of HA. The pH was adjusted to 5.5 ± 0.2. Following 1 h of stirring, the pH was raised to 7.2 ± 0.2 through 5 M NaOH addition. The conjugating reaction was allowed for 48 h at room temperature before purifying by dialyzing against demineralized water using regenerated cellulose dialysis tubes with nominal MW cut-off of 8-14 kDa for 3 days. Products were freeze-dried and the degree of functionalization was determined via ^1^H NMR (Bruker Fourier 300 NMR spectrometer, US). Deuterium oxide (D_2_O) was used as the solvent for NMR sample preparation.

### 2.3. Preparation of Drug-Free and AA-Loaded HA-pNIPAM Nanogels

HA-pNIPAM nanogels were prepared by a simple sonication method in aqueous solution from three concentrations of the polymer (0.1, 0.15, and 0.25% *w*/*v*). After sonicating for 1 h, the prepared nanogels were settled at 4 °C overnight before drug incubation. Excess amount of AA from 10 mg/mL of ethanolic AA stock solution was added dropwise at a constant stir of 300 rpm using a magnetic stirrer (Glassco 710.DG.0, Glassco Laboratory Equipments Pvt. Ltd., Haryana, India) into the nanogel solution in 1:10 *v*/*v*, incubated at 25 °C for 6 h under light protection. The unloaded AA was discarded by centrifugation at 3000× *g* for 10 min at 4 °C using a microcentrifuge (TOMY, MX-305, Meditop Co., Ltd., Tokyo, Japan). The resulting AA-loaded nanogel formulations were named AA-HA-pNIPAM 0.1, AA-HA-pNIPAM 0.15 and AA-HA-pNIPAM 0.25.

### 2.4. Lower Critical Solution Temperature (LCST) of HA-pNIPAM Nanogels

LCSTs of drug-free nanogels were analyzed by measuring the particle size in the form of temperature trend by dynamic light scattering (DLS) using a Zetasizer (Malvern Nano ZS, Malvern Instruments Ltd., Malvern, UK) with water as the dispersant. The temperature-induced sol–gel transition of nanogels at LCST was observed by the dramatic change in the size of the nanogel without the changes in overall viscosity. To examine the reversible thermoresponse, Z-average sizes of the nanogels were measured without dilution using an automatic controlled temperature program, increasing the temperature from 25–40 °C, followed by decreasing temperature from 40–25 °C at 1 °C/min. The temperature trends were measured by cumulative analysis using Malvern software.

### 2.5. Particle Size and Morphology

The particle size distributions of drug-free and AA-loaded nanogels were measured by DLS and by nanoparticle tracking analysis (NTA) using a NanoSight (Malvern NS300, Malvern Instruments Ltd., Malvern, UK) at 25 °C. Blank nanogels were diluted 2.3 times with ultrapure water (UPW) and AA-loaded nanogels 200 times prior to both size measurements. Morphology of the particles was observed by transmission electron microscopy (TEM) (JEM-1400, JEOL Ltd., Tokyo, Japan). In TEM sample preparation, blank nanogels were not diluted but AA-loaded nanogels were diluted 100 times with UPW prior to negative staining by 0.5% uranyl acetate.

### 2.6. Quantification of AA Using the HPLC Technique

AA quantification was performed by the HPLC technique using an Agilent 1260 Infinity II consisting of a liquid chromatography pump (quaternary pump, G7111A), UV-VIS detector (G7115A), auto sampler (G7129A) (Agilent technologies Inc., Santa Clara, CA, USA) with Chem Station software version E.02.02 and column Luna^®^ C18 with a 250 × 4.5 mm^2^ ID and a C18 guard cartridge column of 4 × 10 mm^2^ (Phenomenex Inc., Torrance, CA, USA). HPLC conditions were isocratic mobile phase; acetonitrile 0.05% H_3_PO_4_ in water (50:50 *v*/*v*); flow rate: 1 mL/min; column temperature: 25 °C; diode array detector wavelength: 206 nm; injection volume: 20 µL; and run time: 10 min. The retention time of AA was observed at 8.2 min. The HPLC method was validated by a linear plot of AA concentrations from 1.25–25 µg/mL using diluent 70% methanol (70:30 *v*/*v* MeOH: H_2_O). Moreover, the method was validated in terms of analytical procedures, specificity, accuracy, intra-day and inter-day precisions, limit of detection (LOD) and limit of quantification (LOQ).

### 2.7. Drug Loading of the Nanogel Formulations

Entrapment efficiency (EE) was studied by the direct method. The nanogel particles and drug-free solution were separated through centrifugal ultrafiltration using a centrifugal filter (Amicon^®^ Ultra 0.5 mL 30 K, Merck Ltd., Darmstadt, Germany). Briefly, 400 µL of AA-loaded nanogels were loaded into the filter and centrifuged at 14,000× *g* for 15 min. The concentrates were completely collected by immediate reverse spin at 1000× *g* for 3 min and thorough rinsing of the centrifuge filter using 60% MeOH. The recovered entrapped drug content was diluted with 70% MeOH, filtered through a 0.22 µm filter and then analyzed by HPLC.

The drug loading efficiency, loading capacity and EE of the nanogels were calculated according to Equations (1)–(3):

(1) $$\mathrm{Loading}\;\mathrm{efficiency}\;\left(\%\right)\;=100\times \frac{\mathrm{Amount}\;\mathrm{of}\;\mathrm{loaded}\;\mathrm{drug}}{\mathrm{Amount}\;\mathrm{of}\;\mathrm{feeding}\;\mathrm{drug}}$$

(2) $$\mathrm{Loading}\;\mathrm{capacity}\;\left(\%\right)\;=100\times \frac{\mathrm{Mole}\;\mathrm{of}\;\mathrm{drug}}{\mathrm{Mole}\;\mathrm{of}\;\mathrm{polymer}}$$

(3) $$\mathrm{EE}\;\left(\%\right)\;=100\times \frac{\mathrm{Amount}\;\mathrm{of}\;\mathrm{entrapped}\;\mathrm{drug}}{\mathrm{Total}\;\mathrm{amount}\;\mathrm{of}\;\mathrm{drug}}\;$$

Therefore, loading efficiency reflects the utilization of drugs in the feed during the AA-loaded nanogel preparation process, loading capacity reflects the capability of a polymer to hold the drug as the ratio per its dry weight, while EE reflects how much the drug can be encapsulated in the nanogel matrix.

### 2.8. Stability Study of Drug-Loaded Nanogels

After the preparation, AA-loaded nanogels were kept at temperatures of 4 °C and 25 °C for 180 days in 15 mL conical tubes under light protection. During that time, changes in drug concentration in the nanogel solutions were examined using HPLC at 0, 3, 5, 10, 30, 90 and 180 days.

### 2.9. Cell Culture Condition

L-929 fibroblasts were cultured in DMEM, supplemented with 10% FBS and 100 U/mL penicillin and 100 U/mL streptomycin. The cells were cultivated at 37 °C in a humidified atmosphere with 95% air/5% CO_2_.

### 2.10. Cell Viability Assay

A cell viability study was performed using the PrestoBlue assay. L-929 cells were seeded on a 96-well plate at a density of 10,000 cells/well and incubated at 37 °C for 24 h. Afterwards, the complete culture media were removed and the cells were gently washed with phosphate buffer saline (PBS; pH 7.4). Next, blank nanogels (polymer concentration from 0.1–0.3% *w*/*v*), different AA-loaded nanogel formulations and free AA in dimethyl sulfoxide (DMSO) solution (AA concentrations from 12.5–400 µM) were added (50 µL/well) to the wells in triplicate along with media (100 µL/well) and incubated for another 24 h. Then, the tested solutions were removed. After washing with PBS, the cells were incubated with 10% PrestoBlue reagent for 1 h at 37 °C. The cells without treatment were used as the positive control, while the media containing 10% PrestoBlue reagent without cells were used as the blank. Fluorescent intensity was measured at 560/590 nm (Ex/Em) using a microplate reader (CLARIOstar, BMG LABTECH Ltd., Ortenberg, Germany). Viability (%) was calculated according to Equation (4):

(4) $$\mathrm{Viability}\;\left(\%\right)\;=100\times \frac{\mathrm{Sample}\;\mathrm{intensity}-\mathrm{Blank}\;\mathrm{intensity}}{\mathrm{Positive}\;\mathrm{control}\;\mathrm{intensity}-\mathrm{Blank}\;\mathrm{intensity}}$$

### 2.11. Single-Dose Toxicity Study in Rat Model

#### 2.11.1. Experimental Animals

Single-dose oral acute toxicity study of HA-pNIPAM and AA-HA-pNIPAM was performed with the dose of AA based on the cell viability trial. A total of 18 female BrlHan:WIST@Jcl (GALAS) rats (approximately 10 weeks old and weighing 177–196 g) were purchased from Nomura Siam International, Thailand, and housed at the Chulalongkorn University Laboratory Animal Center (CULAC). Briefly, rats were kept under a 12 h light-dark cycle at 22 ± 3 °C with 40–60% humidity for 1 week for adaptation with free access to pellet food and water. Animal welfare and experimental procedures were carried out in strict accordance with the OECD Guidelines for a single-dose acute oral toxicity study and all experimental protocols were approved by the Chulalongkorn University Animal care and Use Committee (CU-ACUC). The animals were randomly allocated into three groups (*n* = 6 per group). Group 1 was the control and was administered with UPW, and Groups 2 and 3 were the experimental groups and were administered with HA-pNIPAM (0.5 mg/kg body weight) and AA-HA-pNIPAM (0.5 mg HA-pNIPAM + 6 µg AA/ kg body weight) by oral gavage, respectively.

#### 2.11.2. Clinical Pathology

Blood samples for hematological and clinical chemistry analyses were taken from animals at termination. For hematological analyses, blood samples were collected into K3 EDTA tubes (Vet and Vitro Lab Group, Bangkok, Thailand) and analyzed with a hematology analyzer (Mindray, BC-5000 vet). Blood samples for biochemical studies were collected into lithium heparin tubes and analyzed using a blood analyzer (Dirui, CS400). For biochemical analyses, a comprehensive diagnostic profile and mammalian liver profile were performed.

#### 2.11.3. Organ Weights and Histopathological Studies

The following organs were weighed at necropsy: brains, livers, kidneys, and spleens. For histopathological studies, all rats were euthanized on day 14 by using CO_2_ and tissues were surgically removed and stored in 10% formalin. For microscopic analysis, fixed tissues were dehydrated by treating with 70%, 80%, and absolute alcohol and embedded in paraffin block. Thin sections (5 µm) were made using a microtome (Thermo scientific, Shandon Finesse 325, Waltham, MA, USA) before staining with hematoxylin and eosin (H&E) dye. The stained sections were observed with a light microscope (Nikon Eclipes E600, Nikon, Tokyo, Japan) and imaged with a Nikon Digital Camera DXM 1200F (Nikon Eclipes E600, Nikon, Tokyo, Japan).

### 2.12. Statistical Analysis

All experiments were repeated at least three times. Results are expressed as mean ± SD. Statistical analysis was performed for evaluating statistical differences in loading amount, loading efficiency, loading capacity, entrapment efficiency, cell viability, % drug loading in the stability study and in-vivo study by one-way ANOVA, while the size measurement comparison was performed by paired t-test using SPSS 17 software. *p* value < 0.05 was considered as the level of significance.

## 3. Results and Discussion

### 3.1. Preparation of Drug-Free and AA-Loaded Nanogels

**Figure 1:**
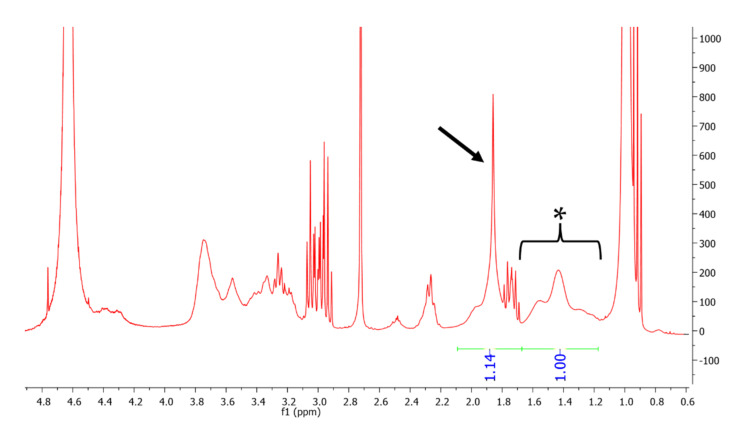
^1^H NMR spectrum of HApNIPAM copolymer showing 4.88% grafting of pNIPAM. The asterisk (1.1–1.7 ppm) represents the peak of 3 protons from the acetyl group of HA and that of 1 proton from the pNIPAM chain, and the arrow (1.7–2.1 ppm) represents the peak of 2 protons from the pNIPAM chain.

The HA-pNIPAM copolymer was prepared by conjugating pNIPAM to the backbone of the HA polymer. The nanogel particles were formed by sonication, introduction of AA into the nanogel solution and subsequent centrifugation for removing insoluble AA. The production of the grafted polymer was analyzed using ^1^H NMR. NMR spectra of unmodified HA and pNIPAM from previous studies were used as references [45,47]. As shown in Figure 1, the degree of grafting was calculated to be 4.88%. Polymer concentrations of 0.1, 0.15 and 0.25% *w*/*v* were used to form nanogel formulations designated as HA-pNIPAM 0.1, HA-pNIPAM 0.15 and HA-pNIPAM 0.25, respectively. Each formulation was incubated with an excess amount of AA at 25 °C for 6 h followed by centrifugation.

### 3.2. LCST Behavior

**Figure 2:**
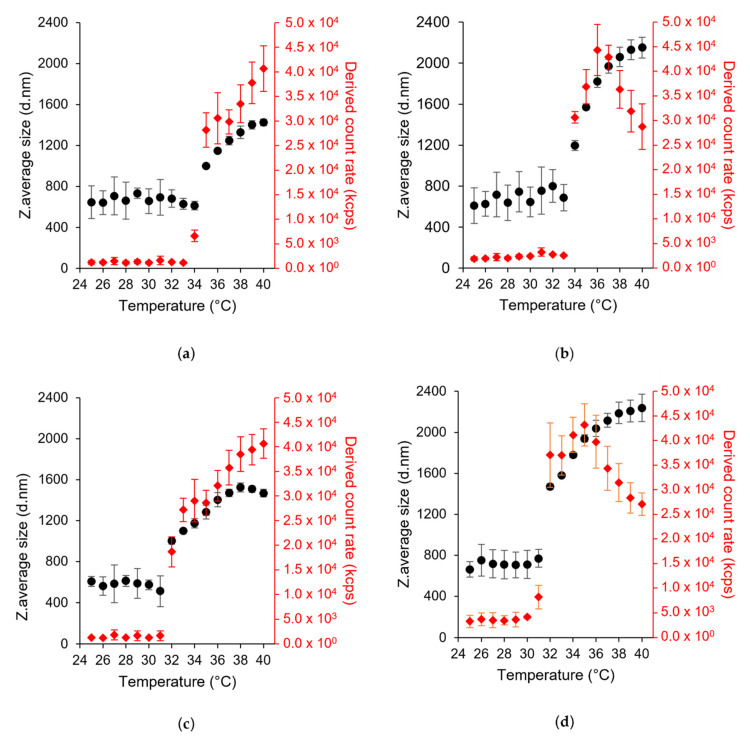
Particle size (left y-axis) and light scattering intensity (in kcps, kilo count per second) (right y-axis) as a function of temperature in DLS, (**a**,**b**) HA-pNIPAM 0.1 and HA-pNIPAM 0.25 when heating from 25 °C to 40 °C, (**c**,**d**) HA-pNIPAM 0.1 and HA-pNIPAM 0.25 when cooling from 40 °C to 25 °C, respectively (mean ± SD, *n* = 3). The first temperature point showing the significant change in the size of the nanogels indicates LCST.

DLS was used to measure the LCST of the HA-pNIPAM nanogels using the temperature trend system. In our study, both HA-pNIPAM 0.1 and HA-pNIPAM 0.25 showed a reversible thermoresponse as shown in Figure 2. The sizes of both were not significantly different in the size range 600–750 nm below LCST. However, the size of HA-pNIPAM 0.25 became significantly higher than that of HA-pNIPAM 0.1 above the LCST (*p* = 0.000). This shows that higher polymer concentrations had higher size changes above the LCST. This might be due to the greater availability of pNIPAM and HA concentrations responsible for the higher degree of size changes.

LCST values of HA-pNIPAM 0.1 and HA-pNIPAM 0.25 were 35 °C and 34 °C in the heating cycle (25–40 °C) (Figure 2a,b). They are 3 °C and 2 °C higher than the original LCST of pNIPAM (LCST = 32 °C). Therefore, hydrophilic HA polymer could increase the LCST of pNIPAM. HA-pNIPAM polymer concentration has a significant influence on the LCST in the sol to gel transition. A lower polymer concentration tends to have lower availability of pNIPAM and vice versa. At a grafting degree of 4.88% of pNIPAM to HA, low polymer concentration resulted in an LCST higher than the high polymer concentration. It can be said that higher energy is required to transit sol to gel in a low pNIPAM content compared with a high HA concentration. This agrees with the statements that hydrophobic components tend to decrease LCST [48] and hydrophilic comonomers could increase LCST [49]. However, LCST in the cooling cycle was reduced to 32 °C in both nanogel solutions (Figure 2c,d). This kind of phase transition also occurred in HA-grafted chitosan-grafted pNIPAM polymer (HA-CPN) measured using a UV/Vis spectrophotometer. In their study, the addition of HA resulted in a slightly higher LCST (30.3 °C) during gel forming; however, there was a lower LCST (27.8 °C) during gel melting compared to the LCST of pNIPAM-COOH and CPN polymers due to the hydrophilic groups such as carboxylic acid (-COOH) and hydroxyl (–OH) groups. Compared with pNIPAM-COOH and chitosan-grafted pNIPAM (CPN), gelation of more complicated molecules, HA-CPN, might lead to more complicated physical entanglement resulting in a longer gel formation time and more liquefaction time for high-molecule weight polymers to rearrange and disentangle inter- and intra-molecular chains [41].

Moreover, DLS is a powerful tool for accessing the detailed information about the size distribution of aggregates. The hydrodynamic size of the nanogel showed a lower polydispersity index (PDI) value at and above LCST. In the heating cycle, HA-pNIPAM 0.1 has a PDI of 0.45 at 25 °C, 0.11 at 35 °C (LCST) and 0.06 at 40 °C. In addition, HA-pNIPAM 0.25 has a PDI of 0.58 at 25 °C, 0.19 at 34 °C (LCST) and 0.24 at 40 °C. In the cooling cycle, the PDI is 0.67, 0.22 and 0.04 for HA-pNIPAM 0.1 and 0.58, 0.22 and 0.18 for HA-pNIPAM 0.25 at 25 °C, 32 °C (LCST) and 40 °C, respectively. The peak of scattered light intensity increased at and above the LCST (Figure 2). Thus, it agrees with the Z-average size of the nanogel. One hypothesis is that some HA-pNIPAM polymer could be released and re-assembled into new nanoparticles along the temperature increase from LCST because of the progressive hydrophobic transition of HA-pNIPAM in the nanogel formulation [50]. Therefore, the continuous increases of the scattered light intensity and the particle sizes and the additional decrease in the size distribution at and above the LCST supported the combinational events of the expansion of the nanogels and the formation of new particles at and above the LCST.

However, in HA-pNIPAM 0.25, the scattered light intensity began to decrease when the temperature reached 37 °C in the heating cycle and 35 °C in the cooling cycle (Figure 2b,d). In HA-pNIPAM 0.1, the maximum size in the temperature trend is less than 1600 nm. Compared to HA-pNIPAM 0.1, HA-pNIPAM 0.25 showed higher size changes.

### 3.3. Size and Morphology of the Nanogel Formulations

**Figure 3:**
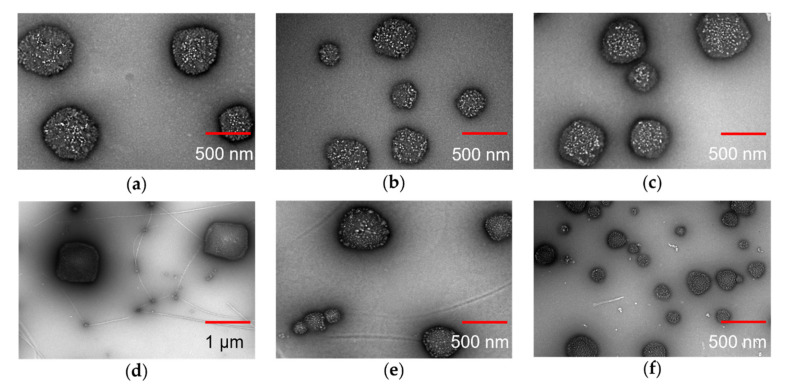
TEM images of assembled nanogels. (**a**) HA-pNIPAM 0.1, (**b**) HA-pNIPAM 0.15, (**c**) HA-pNIPAM 0.25, (**d**) AA-HA-pNIPAM 0.1 (4 °C), (**e**) AA-HA-pNIPAM 0.15 (4 °C) and (**f**) AA-HA-pNIPAM 0.25 (4 °C). Scale bars indicate 500 nm except 1 µm for (**d**).

As shown in Figure 3, TEM images showed that the particles have well-defined spherical shapes except the AA-HA-pNIPAM 0.1 nanogel with the rectangular shape. The sizes are approximately 280–600 nm in the HA-pNIPAM nanogels (Figure 3a–c), 200–700 nm in the AA-HA-pNIPAM 0.15 (Figure 3e) and 60–670 nm in the AA-HA-pNIPAM 0.25 (Figure 3f), while the AA-pNIPAM 0.1 nanogel showed extremely large (600–900 nm) and small sizes (20–70 nm) (Figure 3d).

There are no size changes among HA-pNIPAM 0.1, 0.15 and 0.25 nanogel formulations compared to both TEM analysis (Figure 3a–c) and DLS measurements (Table 1). The size of the nanogels depends on several factors such as the degree of substitution of the hydrophobic moiety in the polymer [51] and the solvent [52,53] for nanogel preparation. Even though another study showed that the polymer concentration may have influences on the particle size of the nanogel [54], such effect was not observed in our study. That might be because the range of the polymer concentrations is narrow.

As shown in Table 1, the sizes of both drug-free and drug-loaded nanogels measured by DLS were significantly different from those measured by NTA except in HA-pNIPAM 0.25. PDI of the nanogels was found to be 0.19–0.41 by DLS. The great variations in particle size between Z-average size by DLS and mean size by NTA might be due to the polydispersity of the nanoparticles. The presence of a few large particles could interfere with the diameter size detection due to their contribution to a more scattered signal, resulting in overestimation of the size. Close agreement of size based on NTA and DLS can be achieved in monodispersed particles [55,56]. However, TEM data supported the size range observed in both DLS and NTA.

Moreover, to obtain the optimal particle number for NTA and TEM analysis, AA-loaded nanogels were diluted 200 and 100 times with UPW while drug-free nanogels were only diluted 2.3 times and there was no dilution in NTA and TEM. Therefore, the number of nanogel particles was obviously a lot higher in AA-loaded nanogels compared with drug-free nanogels. The size, shape and the number of the nanogels are different after drug loading compared to TEM and NTA studies. This observation could be caused by re-assembling of the polymer under mechanical force during drug incubation and the formation of new particles after the introduction of hydrophobic AA molecules. The hydrophobic interaction of AA with polymer chains could not only induce the aggregation of the HA-pNIPAM polymer [57] resulting in newly formed particles but also make the preformed nanogels more compact and smaller in size [45].

### 3.4. Drug Loading in Nanogels

As shown in Table 2, AA-HA-pNIPAM 0.15 and 0.25 nanogels had significantly higher EE % than AA-HA-pNIPAM 0.1 nanogel. It was shown that EE % is likely to be higher in nanoparticles prepared from a higher polymer concentration [58]. However, loading amount, drug loading efficiency and loading capacity were significantly higher in the nanogel assembled from lower polymer concentrations. As from the TEM analysis, AA was not completely soluble in the AA-HA-pNIPAM 0.1 nanogel solution. The filaments are dispersed and stabilized by the small nanogel particles. This could explain why a lower polymer concentration has a higher drug loading in this study. The concentration of AA loaded in water prepared from the same drug loading procedures as in the nanogel was found to be 3.98 ± 0.14 µM. The nanogels could increase AA loading of approximately 400, 370 and 250 folds in AA-HA-pNIPAM 0.1, 0.15 and 0.25 nanogels, respectively. Therefore, the nanogel formulations could improve the bioavailability of AA by increasing its aqueous solubilization.

### 3.5. Stability Study

**Figure 4:**
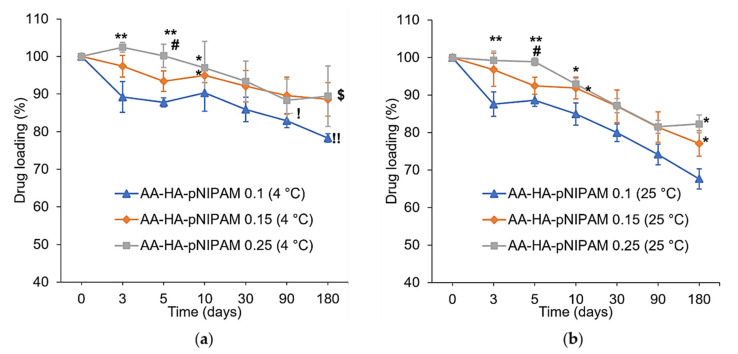
Analysis of % drug loading at (**a**) 4 °C and (**b**) 25 °C/60% RH, over six months (mean ± SD, *n* = 3). * *p* < 0.05, ** *p* < 0.01 vs. AA-HA-pNIPAM 0.1 (4 °C) in (**a**) and AA-HA-pNIPAM 0.1 (25 °C) in (**b**). ^#^ *p* < 0.01 vs. AA-HA-pNIPAM 0.15 (4 °C) in (**a**) and AA-HA-pNIPAM 0.15 (25 °C) in (**b**). ^!^ *p* < 0.05, ^!!^ *p* < 0.01 vs. AA-HA-pNIPAM 0.1 (25 °C). ^$^ *p* < 0.05 vs. AA-HA-pNIPAM 0.15 (25 °C).

The stability study of AA-loaded nanogels was performed at two temperatures (4 °C and 25 °C) as shown in Figure 4. AA-HA-pNIPAM 0.1 has significantly less % drug loading than AA-HA-pNIPAM 0.25 at day 3, 5 and 10 at both temperatures. The same event happens again at day 180 only at 25 °C where AA-HA-pNIPAM 0.1 has less % AA loading than the other two formulations. Additionally, AA-HA-pNIPAM 0.1 at 4 °C has % drug loading higher than that at 25 °C since day 90. This shows that nanogel prepared from a lower polymer concentration is less stable compared with those prepared from a higher polymer concentration. At the same time, the nanogels were more stable at 4 °C, at which % of drug loading remains 78.29 ± 1.15 %, 88.06 ± 4.45% and 89.44 ± 8.01 % in AA-HA-pNIPAM 0.1, 0.15 and 0.25 nanogels, respectively, after 180 days. Similar to AA-HA-pNIPAM 0.1, AA-HA-pNIPAM 0.15 has significantly less % drug loading than AA-HA-pNIPAM 0.25 at day 5 at both temperatures. Again, at day 180, AA-HA-pNIPAM 0.15 at 4 °C shows significantly higher % drug loading than that at 25 °C. The results gave the same conclusion as described above. Furthermore, the stability profiles of the nanogels are different. Lower polymer concentration at both temperatures resulted in the abrupt decrease in % drug loading at the early days of storage (day 3, 5 and 10) followed by a gradual decrease. Therefore, nanogels prepared from a low polymer concentration could not tolerate short-term storage. From the visual analysis of the nanogel solutions, higher polymer concentrations resulted in higher suspension of the polymer at the bottom of the containers after 30 days of storage. The polymer suspension could be redispersed and stable in the solution with no further precipitation for at least one month. However, drug loading was measured without prior shaking in all three formulations over the period of 180 days.

### 3.6. Cell Viability Study

**Figure 5:**
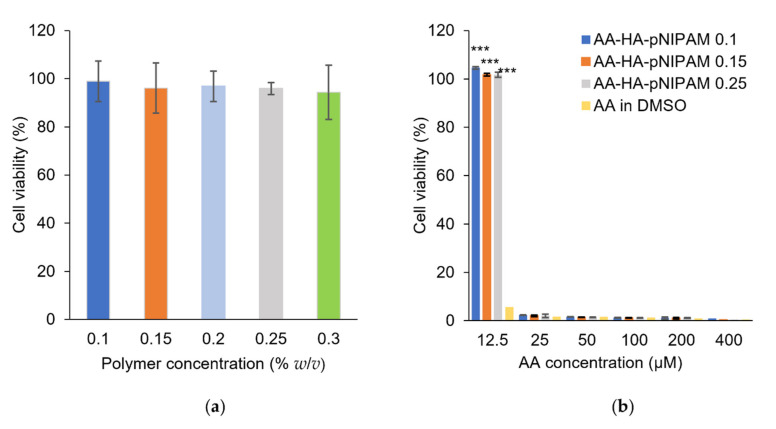
PrestoBlue assay showing the relative non-toxic effects of (**a**) drug-free nanogel, and (**b**) AA-loaded HA-pNIPAM nanogels to L-929 cells (mean ± SD, *n* = 3). *** *p* < 0.001 vs. AA in DMSO.

A PrestoBlue viability assay was performed using drug-free nanogels, drug-loaded nanogels and AA in DMSO solution and the results are shown in Figure 5. Cytotoxic effects were not observed in nanogels without the drug (Figure 5a). AA concentration at 12.5 µM in DMSO expressed cytotoxicity in L-929 cells; however, the same AA concentration loaded in nanogels had no toxic effect to the cells (Figure 5b). This result suggested that the nanogels could improve the biocompatibility of the AA towards L-929 cells at an AA concentration of 12.5 µM. Meanwhile, dose-dependent cytotoxicity was observed in AA-loaded nanogels when AA concentration was higher than 12.5 µM.

### 3.7. Single-Dose Toxicity Study in Rat Model

#### 3.7.1. Body Weight

**Figure 6:**
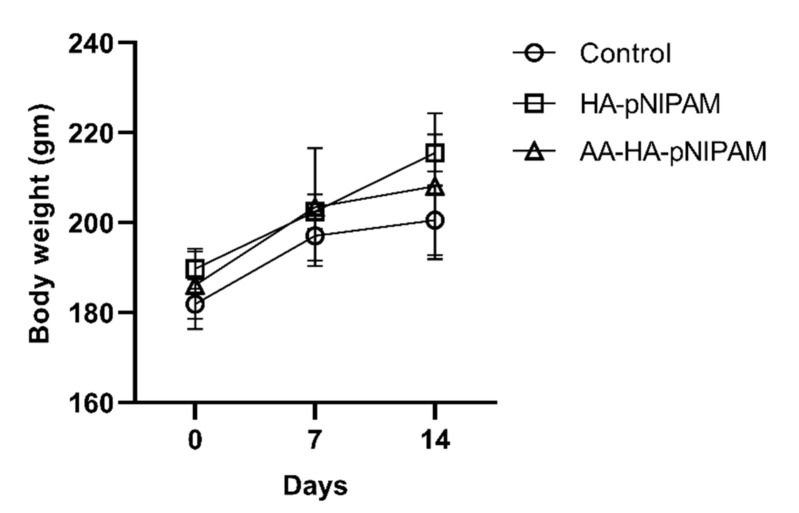
Effect of single-dose treatment on average body weight of rats (mean ± SD, *n* = 5 in control and *n* = 6 in HA-pNIPAM and AA-HA-pNIPAM).

The animals were weighed on a day before the treatments, day 7 and day 14. A slight body weight increase was observed in each group as shown in Figure 6. Statistical analysis of animal body weights showed no significant differences between control (UPW) and treatment groups (HA-pNIPAM and AA-HA-pNIPAM).

#### 3.7.2. Clinical Pathology

**Figure 7:**
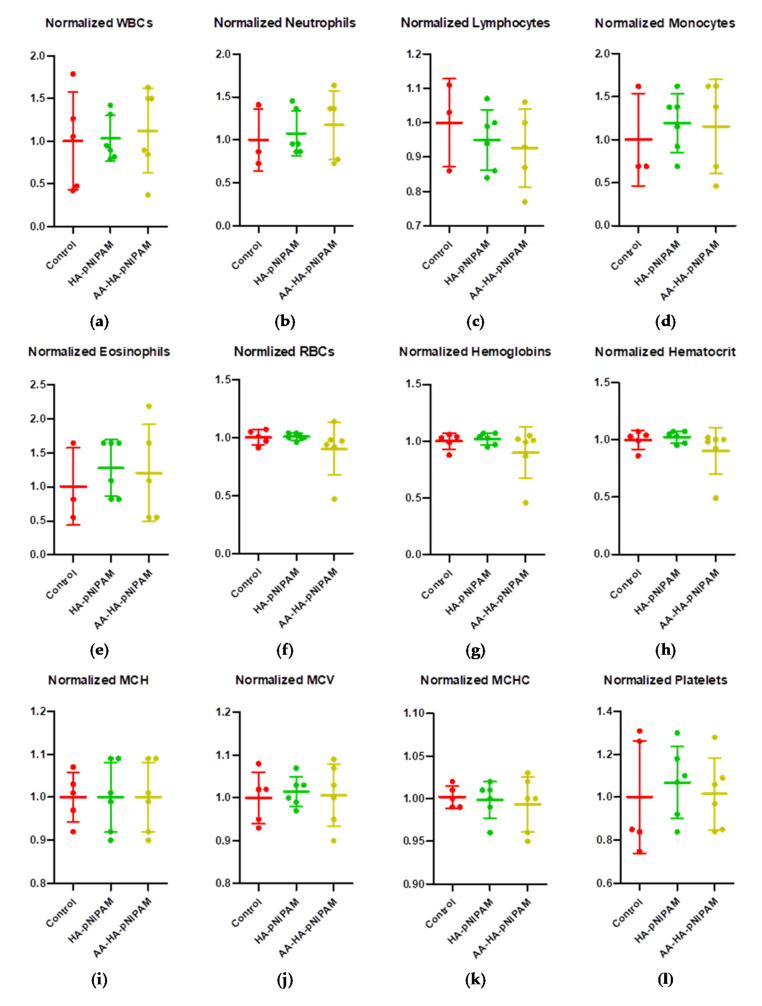
Hematological changes in rats treated with control, HA-pNIPAM and AA-HA-pNIPAM. The hematological parameters analyzed were (**a**) white blood cells (WBCs), (**b**) neutrophils, (**c**) lymphocytes, (**d**) monocytes, (**e**) eosinophils, (**f**) red blood cells (RBCs), (**g**) hemoglobin, (**h**) hematocrit, (**i**) mean cell hemoglobin (MCH), (**j**) mean corpuscular volume (MCV), (**k**) mean corpuscular hemoglobin concentration (MCHC) and (**l**) platelets.

**Figure 8:**
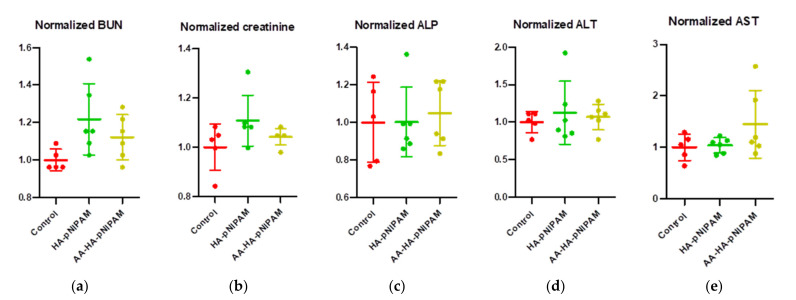
Biochemical analyses from the blood samples of rats after treatment with control, HA-pNIPAM and AA-HA-pNIPAM. The biochemical parameters analyzed were (**a**) blood urea nitrogen (BUN), (**b**) creatinine, (**c**) alkaline phosphatase (ALP), (**d**) alanine aminotransferase (ALT) and (**e**) aspartate aminotransferase (AST).

We further investigated whether treatments cause a change in hematological parameters, including white blood cells (WBCs), neutrophils, lymphocytes, monocytes, eosinophils, red blood cells (RBCs), hemoglobin, hematocrit, mean cell hemoglobin (MCH), mean corpuscular volume (MCV), mean corpuscular hemoglobin concentration (MCHC) and platelets. Figure 7 demonstrates that there were no significant changes in such parameters after treatments. Moreover, we further observed the important biochemical parameters, including blood urea nitrogen (BUN), creatinine, alkaline phosphatase, alanine aminotransferase (ALT) and aspartate aminotransferase (AST). Consistent with hematological parameters, no significant alteration could be detected in the biochemical parameters of the treatment groups (Figure 8). These results strongly support the safety of this platform.

#### 3.7.3. Organ Weight and Histopathological Findings

**Figure 9:**
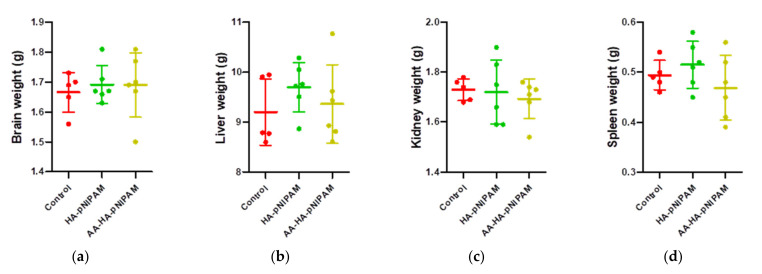
Organ weights of rats treated with control, HA-pNIPAM and AA-HA-pNIPAM. The organs weighed at necropsy included (**a**) brains; (**b**) livers; (**c**) kidneys and (**d**) spleens.

**Figure 10:**
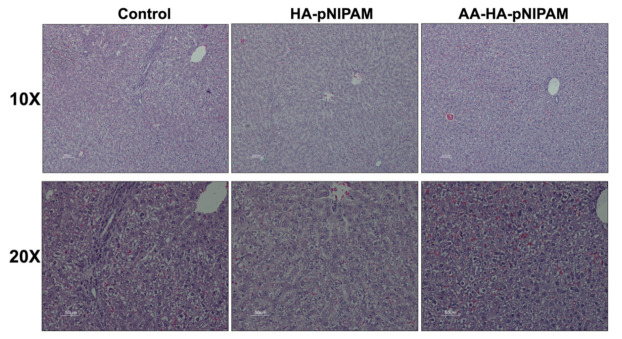
Microscopic images of livers from rats treated with UPW, HA-pNIPAM and AA-HA-pNIPAM. Hematoxylin and eosin (H&E) staining; 10× and 20× magnification.

**Figure 11:**
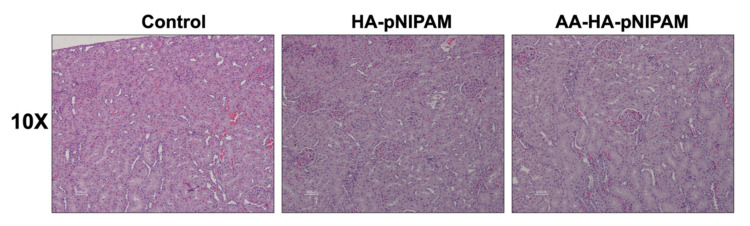
Microscopic images of kidneys from rats treated with UPW, HA-pNIPAM and AA-HA-pNIPAM. Hematoxylin and eosin (H&E) staining; 10× magnification.

Figure 9 shows no significant differences in the organ weights between control and experimental groups. Figure 10 shows marked glycogen accumulation in the liver, and Figure 11 shows mild congestion in the kidneys in all three groups. The histological scores, classified into 0 (no change recorded), 1 (mild for <33% of tissues affected), 2 (moderate for 33–66% of tissues affected) and 3 (marked for >66% of tissues affected), were not significantly different among the three animal groups.

To summarize, there were no significant differences in the body weight, organ weights, hematological parameters, clinical chemistry values and histopathological examination from the experimental groups as compared to the control group. Therefore, our in vivo information suggested that HA-pNIPAM and AA-HA-pNIPAM were non-toxic to the animals at the applied dose, supporting the safety of the nanogel system.

## 4. Conclusions

Nanogels were formulated by a simple sonication method using a non-toxic and biocompatible polymer at three concentrations (0.1, 0.15 and 0.25% *w*/*v* HA-pNIPAM polymer) in water. In this study, an HA-pNIPAM nanogel system was developed to overcome the poor solubility issue of a promising bioactive candidate, AA. The experiment was designed to formulate an HA-based nanogel delivery system prepared from the polymer, synthesized by the modification of HA with pNIPAM. The properties of the nanogel prepared from different polymer concentrations on the drug loading and stability were characterized. AA loading was higher in nanogels prepared from lower polymer concentrations. However, the stability of the nanogel formulation was higher in AA-HA-pNIPAM 0.25 according to % drug loading over 180 days. The cell viability assay proved no cytotoxicity effect of drug-free nanogels. Additionally, drug-loaded nanogels were cytocompatible up to 12.5 µM of AA. Furthermore, no evident toxicity was observed in rats treated with both HA-pNIPAM (0.5 mg/kg body weight) and AA-HA-pNIPAM (HA-pNIPAM 0.5 mg and AA 6 µg/kg body weight). Therefore, this nanogel system has great potential for improving water solubility and stability of poor-solubility drugs and its biocompatibility ensures the safety of the nanogel.

## Figures and Tables

**Table 1:** The comparison of particle sizes of the nanogels using DLS and NTA at 25 °C (mean ± SD, *n* = 3).

Formulations (Storage Temperature °C)	DLS (Intensity Based)		NTA (Number Based)
	Z-Average Particle (nm)	PDI	Mean Size (nm)
HA-pNIPAM 0.1 (4 °C)	667 ± 198 *	0.28 ± 0.17	443 ± 11
HA-pNIPAM 0.15 (4 °C)	567 ± 83 **	0.34 ± 0.17	461 ± 14
HA-pNIPAM 0.25 (4 °C)	571 ± 61	0.29 ± 0.06	552 ± 27
AA-HA-pNIPAM 0.1 (4 °C)	785 ± 54 ***	0.27 ± 0.12	356 ± 33
AA-HA-pNIPAM 0.15 (4 °C)	653 ± 112 ***	0.26 ± 0.11	378 ± 37
AA-HA-pNIPAM 0.25 (4 °C)	626 ± 62 ***	0.25 ± 0.02	370 ± 19
AA-HA-pNIPAM 0.1 (25 °C)	808 ± 40 ***	0.41 ± 0.12	374 ± 14
AA-HA-pNIPAM 0.15 (25 °C)	806 ± 76 ***	0.38 ± 0.14	419 ± 57
AA-HA-pNIPAM 0.25 (25 °C)	637 ± 120 ***	0.19 ± 0.04	413 ± 10

* *p* < 0.05, ** *p* < 0.01, *** *p* < 0.001 vs. mean size from NTA measurement.

**Table 2:** Loading amount, loading efficiency, loading capacity and EE of the nanogel formulations (mean ± SD, *n* = 3) ^#^.

AA Loaded Nanogels	Loading Amount (mM)	Loading Efficiency (%)	Loading Capacity (%)	EE (%)
AA-HA-pNIPAM 0.1	1.63 ± 0.08 ^a^	79.65 ± 4.48 ^a^	13048.24 ± 733.31 ^a^	86.48 ± 4.79 ^a^
AA-HA-pNIPAM 0.15	1.51 ± 0.04 ^b^	73.61 ± 2.06 ^b^	8039.06 ± 225.10 ^b^	98.64 ± 3.94 ^b^
AA-HA-pNIPAM 0.25	1.00 ± 0.19 ^c^	49.10 ± 10.90 ^c^	3206.26 ± 706.50 ^c^	101.15 ± 9.34 ^b^

The different superscript letters ^a,b,c^ refer to statistically significant differences between each set of data (*p* < 0.05). ^#^ The statistical analysis of each parameter is demonstrated in Table S1 in the Supplementary Materials.

## Data Availability

Data are available upon request.

## Supplementary Materials

The following are available online at https://www.mdpi.com/article/10.3390/polym13234071/s1, Table S1: Statistical analysis of loading amount, loading efficiency, loading capacity and entrapment efficiency of the nanogel formulations (mean ± SD, *n* = 3).
