# Supplementary material for: In Vivo Biocompatible Self-Assembled Nanogel Based on Hyaluronic Acid for Aqueous Solubility and Stability Enhancement of Asiatic Acid
Source: Polymers (Basel). 2021 Nov 23;13(23):4071. doi: 10.3390/polym13234071 (PMC8659171; doi:10.3390/polym13234071)
Supplement: Supplementary file 1 [file polymers-13-04071-s001.zip › polymers-1416133-supplementary.pdf]

polymers

MDPI

Supplementary File

In Vivo Biocompatible Self-Assembled Nanogel Based on Hyaluronic Acid for Aqueous Solubility and Stability Enhancement of Asiatic Acid

Yu Yu Win, Penpimon Charoenkanburkang, Vudhiporn Limprasutr, Ratchanee Rodsiri, Yue Pan, Visarut Buranasudja, Jittima Amie Luckanagul*

Supplementary information

Dependent variables	Independent variables	Independent variables	Mean difference	*p* value
Loading amount (mM)	AA-HA-pNIPAM 0.1	AA-HA-pNIPAM 0.15	0.124**	0.005**
		AA-HA-pNIPAM 0.25	0.625***	< 0.001***
	AA-HA-pNIPAM 0.15	AA-HA-pNIPAM 0.25	0.510***	< 0.001 ***
Loading efficiency (%)	AA-HA-pNIPAM 0.1	AA-HA-pNIPAM 0.15	6.041**	0.005**
		AA-HA-pNIPAM 0.25	30.546***	< 0.001***
	AA-HA-pNIPAM 0.15	AA-HA-pNIPAM 0.25	24.505***	< 0.001***
Loading capacity (%)	AA-HA-pNIPAM 0.1	AA-HA-pNIPAM 0.15	5008.641***	< 0.001 ***
		AA-HA-pNIPAM 0.25	9841.981***	< 0.001***
	AA-HA-pNIPAM 0.15	AA-HA-pNIPAM 0.25	4832.804***	< 0.001***
Entrapment efficiency (%)	AA-HA-pNIPAM 0.1	AA-HA-pNIPAM 0.15	-12.159***	< 0.001***
		AA-HA-pNIPAM 0.25	-14.679**	0.002**
	AA-HA-pNIPAM 0.15	AA-HA-pNIPAM 0.25	-2.510	0.811

**$p < 0.01$, ***$p < 0.001$ vs. between mean values of two independent variables.

Table S1: Statistical analysis of loading amount, loading efficiency, loading capacity and entrapment efficiency of the nanogel formulations (mean ± SD, $n = 3$).
